# Macrophage Polarization in the Development and Progression of Ovarian Cancers: An Overview

**DOI:** 10.3389/fonc.2019.00421

**Published:** 2019-05-22

**Authors:** Huiyan Cheng, Zhichao Wang, Li Fu, Tianmin Xu

**Affiliations:** ^1^Department of Gynecology and Obstetrics, The Second Hospital of Jilin University, Changchun, China; ^2^Department of Gynecology and Obstetrics, The First Hospital of Jilin University, Changchun, China; ^3^Department of Pediatric Surgery, The First Hospital of Jilin University, Changchun, China

**Keywords:** macrophage polarization, ovarian cancer, microenvironment, M1/M2, TAMs, exosomes, epigenetic

## Abstract

Ovarian cancer is the most lethal gynecological malignancy worldwide. Most patients are diagnosed at late stages because of atypical symptoms and the lack of effective early diagnostic measures. The mechanisms underlying the oncogenesis and development of ovarian cancer are not clear. Macrophages, immune cells derived from the innate immune system, have two states of polarization (M1 and M2) that develop in response to different stimuli. The polarization and differentiation of macrophages into the cancer-inhibiting M1 and cancer-promoting M2 types represent the two states of macrophages in the tumor microenvironment. The interaction of polarized macrophages with cancer cells plays a crucial role in a variety of cancers. However, the effects of macrophage M1/M2 polarization on ovarian cancer have not yet been systematically and fully discussed. In this review, we discuss not only the occurrence, development and influences of macrophage polarization but also the association between macrophage polarization and ovarian cancer. The polarization of macrophages into the M1 and M2 phenotypes plays a pivotal role in ovarian cancer initiation, progression, and metastasis, and provides targets for macrophage-centered treatment in the cancer microenvironment for ovarian cancer therapy. We also addressed the regulation of macrophage polarization in ovarian cancer via noncoding RNAs, exosomes, and epigenetics.

## Introduction

Ovarian cancer is the most lethal malignancy of the female reproductive tract, and its mortality rate is reported to be the fifth highest among all female cancers (1). The pathogenesis and development of ovarian cancer is associated with various biological and molecular factors, dysfunctional expression or mutation of genes, dysregulation of host immune responses, ovulation frequency, activation of oncogenes or inactivation of suppressor genes, reactions to growth factors, and cytokines in the tumor microenvironment (TME), etc.(2). The progression-free survival (PFS) and overall survival (OS) rates of ovarian cancer patients tend to be poor due to the lack of early testing methods. Seventy percent of ovarian cancer patients will eventually experience recurrence and develop chemoresistance, although most patients accept effective treatments, including cytoreductive surgery and taxane/platinum-based chemotherapy (3). Macrophages are important innate immune system cells that have many physiological functions, and tumor-associated macrophages (TAMs) exist in the cancer microenvironment and influence the formation, growth, and metastasis of cancers by interacting with cancer cells (4). With different stimuli, macrophages can be polarized into classically activated M1 macrophages or alternatively activated M2 macrophages. In cancers, TAMs are considered M2-like and support almost all hallmarks of cancer by producing a large number of growth factors, extracellular matrix (ECM) remodeling molecules, and cytokines to regulate cancer growth, migration and angiogenesis (5). According to previous reports, M2 macrophage polarization is associated with hepatoma (6), prostate carcinoma (7), colon cancer (8), pancreatic cancer (9), thyroid cancer (10), and brain tumors (11), among others.

## Macrophages and Macrophage Polarization and Classification

Macrophages, which are present in almost all tissues and can infiltrate infected or damaged tissue, were discovered by Metchnikoff in 1908 (12). Monocytes develop from embryonic hematopoietic precursors during fetal development and from the stem cells of the hematopoietic system in the bone marrow during adult life (13). Monocytes migrate from the blood to tissues and grow into specific macrophages to adapt to local tissues, such as the bones (osteoclasts), kidneys (mesangial cells), central nervous system (microglial cells), connective tissue (histiocytes), alveoli (dust cells), spleen, liver (Kupffer cells), peritoneum, and gastrointestinal tract (14). The TME is composed of fibroblasts, endothelial cells, myofibroblasts, adipose cells, neuroendocrine cells, immune and inflammatory cells, the blood and lymphatic vascular network, extracellular matrix, etc.(15), and macrophages are an immune cell type in the TME. Macrophages isolated from tumors are named TAMs and are similar to macrophages found in developing and regenerating tissues (16). According to the different functional abilities demonstrated in response to stimuli in the microenvironment, macrophages can be divided into two subsets: classically activated M1 macrophages and alternatively activated M2 macrophages (17). In most cases, prognosis is associated with the proportions of the two macrophage subsets (18).

**Figure 1:**
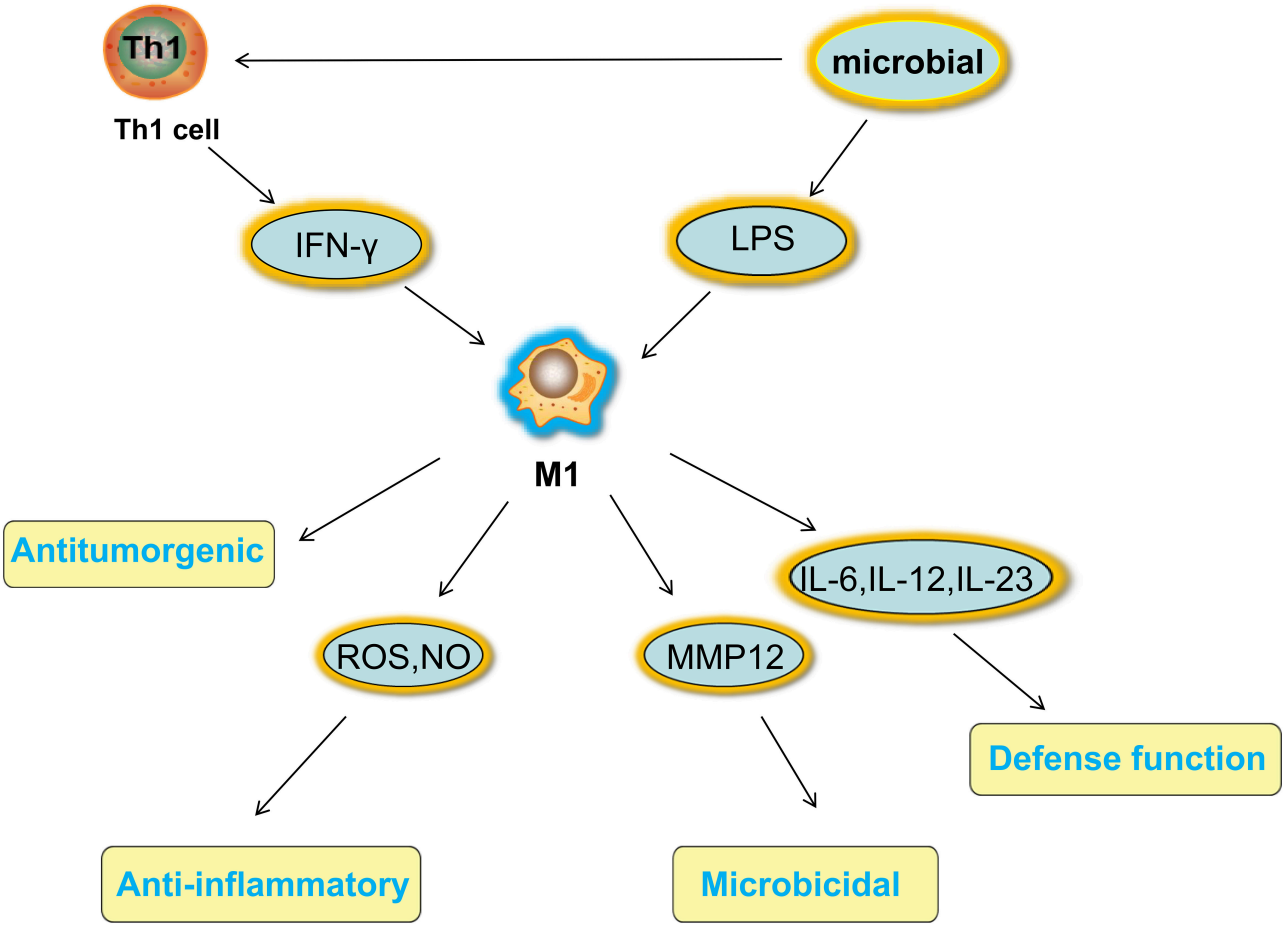
Activated factors and functions of M1 macrophages. Microbial stimuli, such as LPS, and Th1-related cytokines, such as IFN-γ, polarize macrophages into the M1 phenotype. M1 macrophages function in proinflammatory, microbicidal and tumor resistance processes under high production of IL-6 IL-12, and IL-23, high production of toxic intermediates, including NO and ROI, and expression of MMP12. LPS, lipopolysaccharide; IFN, interferon; IL, interleukin; NO, nitric oxide; ROI, reactive oxygen intermediates; MMP12, matrix metalloproteinase 12.

**Figure 2:**
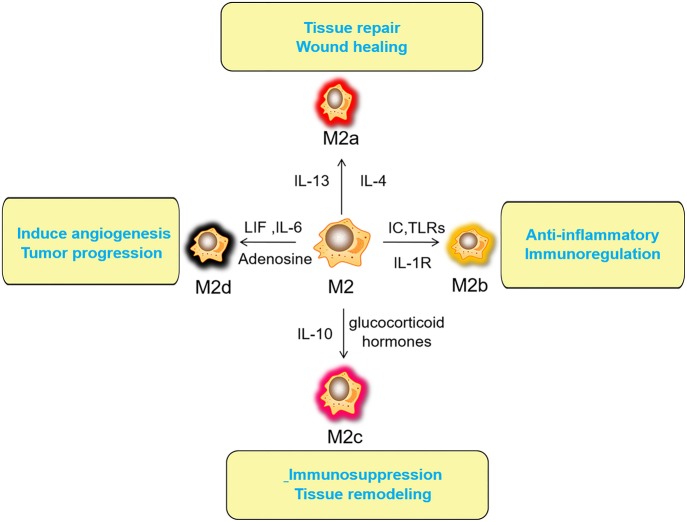
Activated factors and functions of M2 macrophages. The M2a type is induced by IL-4 or IL-13 (promotes tissue repair through the secretion of ECM); the M2b type is induced by exposure to ICs and agonists of TLRs or IL-1R (participates in anti-inflammatory responses and functions in immunoregulation); the M2c type is induced by glucocorticoid hormones and IL-10 (suppresses immune responses and tissue remodeling). M2d (TAMs) macrophages induced by adenosine, LIF and IL-6 are believed to induce angiogenesis to regulate tumor progression and enhance tumor survival. IL, interleukin; ECM, Extracellular matrix; ICs, immune complexes; TLRs, Toll-like receptors; IL-1R, interleukin-1 receptor; TAM, tumor-associated macrophage; LIF, leukemia inhibitory factor.

Macrophages have a strong plasticity and exhibit functional diversity. Macrophages were initially assumed to be involved in antitumor immunity, but they can promote cancer initiation, stimulate angiogenesis, and suppress antitumor immunity during malignant progression (19). The phenotypes of polarized macrophages, including M1 macrophages and M2 macrophages, can be separately altered by the cytokine repertoires of Th1 and Th2 helper cells (20). Microbial stimuli, such as lipopolysaccharide (LPS), and Th1-related cytokines, such as interferon (IFN)-γ, polarize macrophages into the M1 phenotype (21). M1 macrophages function in proinflammatory, microbicidal and tumor resistance processes. M1 macrophages are characterized by the following characteristics: capacity for antigen presentation (22); high production of interleukin (IL)-6, IL-12, and IL-23 (23); high production of toxic intermediates, including nitric oxide (NO) and reactive oxygen intermediates (ROI) (24); and expression of matrix metalloproteinase 12 (MMP12) (25) (Figure 1). Th2 cytokines, such as IL-4 and IL-13, can polarize macrophages into the M2 phenotype, and M2 macrophages function in anti-inflammatory processes, tissue repair and remodeling, parasite clearance, tumor-promoting processes and immunoregulatory processes (26). According to the signal stimuli inducing polarization, M2 macrophages can be classified into three types as follows: the M2a type is induced by IL-4 or IL-13 (promotes tissue repair through the secretion of ECM); the M2b type is induced by exposure to immune complexes (ICs) and agonists of Toll-like receptors (TLRs) or interleukin-1 receptor (IL-1R) (participates in anti-inflammatory responses and functions in immunoregulation); and the M2c type is induced by glucocorticoid hormones and IL-10 (suppresses immune responses and tissue remodeling) (27). TAMs affected by the TME are tumor-promoting cells that play a vital role in cancer growth, invasion and metastasis. TAMs are one type of alternatively activated M2 macrophages (19). Some TAMs appear similar to the M2b phenotype (IL-10^high^, IL-12^low^) (28), while others have been shown to have a tumor necrosis factor (TNF)-α^low^ phenotype similar to the M2c phenotype in some studies on murine and human tumors (26). However, some scholars classify TAMs as M2d macrophages, which express high levels of vascular endothelial growth factor (VEGF) and IL-10. M2d macrophages induced by adenosine, leukemia inhibitory factor (LIF) and IL-6 are believed to induce angiogenesis to regulate tumor progression and enhance tumor survival (29, 30) (Figure 2).

## Expression Markers, Cytokines and Chemokines of Macrophages

Different receptors and distinctive secretion patterns exist for circulating monocytes and tissue macrophages. M1 macrophages highly express MHC-II, IL-1R, TLR2, TLR4, CD80, CD86, and other stimulatory molecules (31). M1 macrophages secrete proinflammatory cytokines, such as TNF-α and IL-1, and some chemokines, such as CCL2, CCL3, CCL5, CXCL8, CXCL9, CXCL10, CXCL11, and CXCL16 (32), and they produce high levels of important inflammatory cytokines, including IL-23, IL-6, and IL-12 (33). M1 macrophages are also associated with the synthesis of reactive oxygen species (ROS) and NO release (34). M2 macrophages express many MHC-II molecules; however, this expression is insufficient for effective antigen presentation (35). They also express high levels of Arginase 1 (Arg1). Arg1 can promote the synthesis of polyamines and stimulate tissue repair, cell growth, collagen formation, etc. (36). M2a macrophages express high levels of surface molecules and receptors, such as CD163, CD23, CD209, Fizz1, Arg1, Ym1/2, IL-4R, FcR, CXCR1, CXCR2, and Dectin-1, in addition to producing CCL17, CCL18, CCL22, and CCL24 (37). M2b macrophages express high levels of the surface molecules CD80 and CD86 and produce TNF-α, CCL1, IL-1, IL-6, and IL-10 (38). M2c macrophages express high levels of surface receptors and molecules, including CD14, CD50, MR, and SR, and produce IL-10, CCL16, CCL18, CXCL13 and transforming growth factor-β (TGF-β) (39). M2d macrophages (TAMs) express high levels of VEGF and CD163 (40), produce cytokines (such as IL-10, IL-12, TNF-α, and TGF-β), and secrete chemokines (CCL5, CXCL10, CXCL16, CCL18) (30) (Table 1).

**Table 1:** The phenotypes, cytokines, and chemokine secretions of M1 macrophage and M2 macrophage.

**Subtypes**	**Expression marker**	**Cytokines**	**Chemokine secretion**
M1	CD80, CD86, MHC-II, CCR7, IL-1R1, TLR2, TLR4	IL-12, IL-1, IL-6, IL-23, TNF	CXCL8, CXCL9, CXL10, CXCL11, CXCL16, CCL2, CCL3, CCL5
M2a	CXCR1, CXCR2, MHC-II, FcR, IL-4R, CD23, CD163, MR (CD206), SR, Ym1/2, Fizz1, Arg-1	IL-10, TGF-β, IL-1R	CCL17, CCL18, CCL22, CCL24
M2b	CD80, CD86, MHC-II	IL-1, IL-6, IL-10, TNF	CCL1
M2c	CD14, CD150, MR (CD26), SR, CCR2	IL-10, TGF-β	CCL16, CCL18, CXCL13
M2d(TAM)	VEGF, CD163	IL-6, IL-10, IL-12, TNF-α, TGF-β	CCL5, CXCL10, CXCL16, CCL18

## Cellular Signaling Pathways

**Figure 3:**
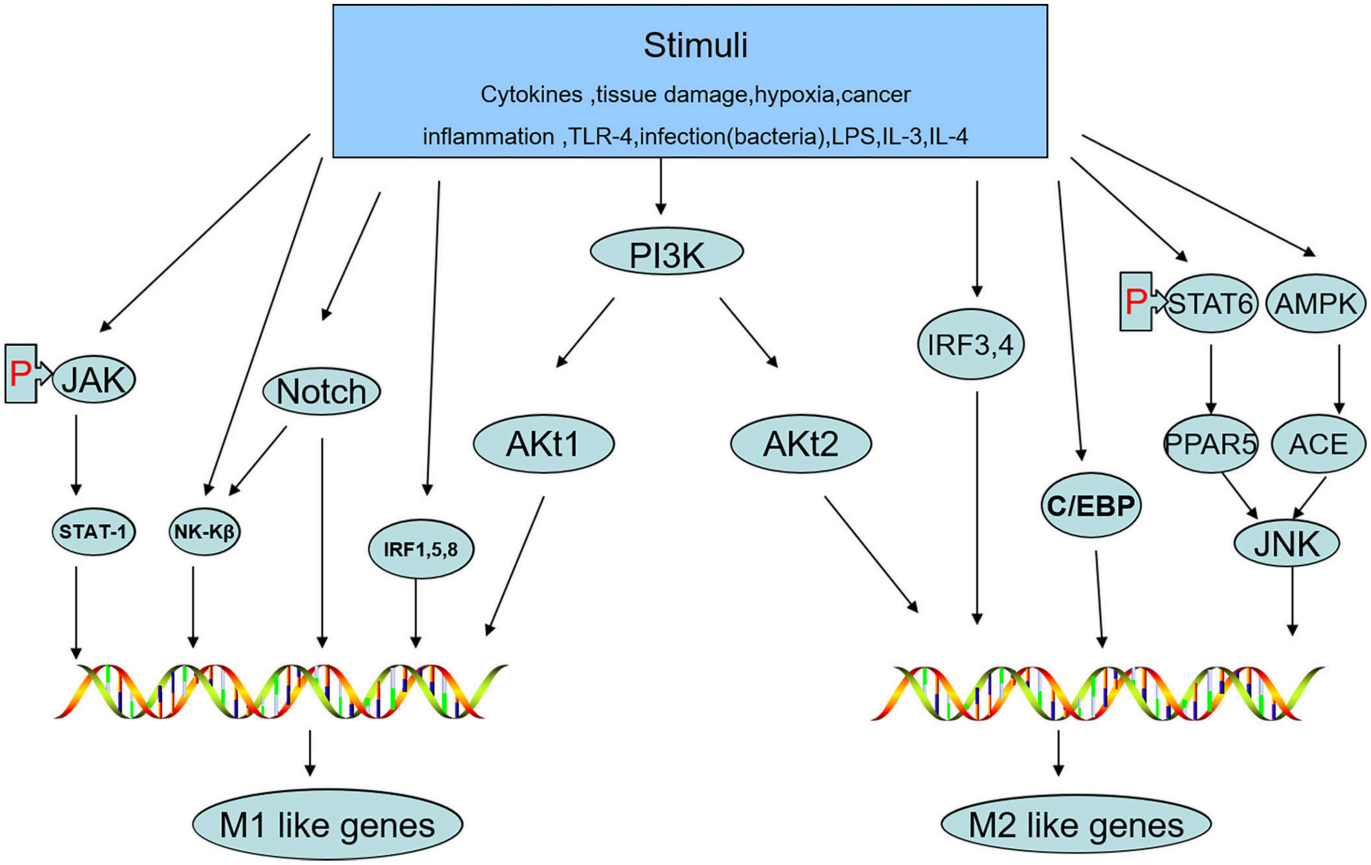
Signaling pathways of macrophage polarization associated with the M1 and M2 phenotypes.

The status of macrophage polarization (M1 and M2) can be further polarized or reversed by cellular signaling pathways. Activation of the JNK signaling pathway polarizes macrophages to the M2 type, while inhibition of JNK activity skews macrophages to the M1 phenotype (41). Th2 cytokines, such as IL-13 and IL-4, whose promoters are regulated by signal transducers and activators of transcription-6 (STAT-6) produce M2-like activation in macrophages by inducing peroxisome proliferator-activated receptor (PPAR) expression (42). AMP-activated protein kinase (AMPK) and factors deriving from adipocytes increase the content of angiotensin-converting enzyme (ACE) in macrophages, polarizing them toward the M2 phenotype (43). Macrophage polarization can be altered by different Akt kinases. Akt1 induces an M1 phenotype, while Akt2 induces an M2 phenotype (44). Notch activation promotes M1 macrophage polarization but inhibits M2 polarization (45). IFN-γ, a potent endogenous macrophage-activating factor, can activate STAT-1 predominantly and induce M1-like macrophage polarization by signaling through the IFN-γ/JAK/STAT-1 pathway (46). PPARγ is a lipid-activated transcription factor in macrophages that can regulate lipid metabolism and the inflammatory response. STAT-6 combines with PPARγ to promote DNA binding and regulate genes, leading to the expression of M2 macrophage markers (47). In addition, interferon regulatory factor (IRF)-1, IRF-5, and IRF-8 are correlated with polarization to the M1 phenotype, while IRF-3 and IRF-4 polarize cells to the M2 phenotype (48). C/EBPβ is a C/EBP family member that has been reported to contribute to macrophage activation and polarization toward the expression of M2-specific genes (49, 50). Because the pathways described above can sometimes interact with each other, studying the signaling pathways of macrophage polarization associated with the M1 and M2 phenotypes plays a crucial role in understanding and creating treatments for the prevention of tumor development (Figure 3).

## Roles of Macrophage Polarization in the Development and Progression of Ovarian Cancer

Macrophages play a crucial role in not only host defense against bacteria, viruses, and parasites but also in defense against tumor cells. Ovarian cancer is the most lethal malignancy of the female reproductive tract. Over 190,000 new cases are diagnosed each year worldwide, and approximately 21,880 new cases and 13,000 deaths occur each year in the United States (51, 52). Most ovarian cancer patients are diagnosed at an advanced stage, and there are three large categories of ovarian cancer: epithelial, germ cell and sex cord. Approximately 80–85% of all ovarian cancers are of the epithelial type, for which the histological subtypes include clear cell, mucinous, endometrioid, and serous carcinoma according to the 2014 WHO classification (53–55). The TME plays an important role in the evolution and progression in cancers. Macrophages are a type of infiltrating immune cell in the TME that have vital physiological and pathological functions. TAMs, which belong to the M2 macrophage phenotype, are related to poor outcomes in solid cancers and play important roles in cancer growth, progression, metastasis, and angiogenesis (56). A high density of CD163+ M2-macrophages is associated with poor prognosis in epithelial ovarian cancer (EOC)(57). High M1/M2 ratios in ovarian tumor tissue are correlated with extended survival (58), while low M1/M2 ratios are correlated with poor overall survival (59).

M2 macrophages may release immunosuppressive factors to support immune evasion in ovarian cancer. For example, macrophages exposed to IL-4, IL-10, and IL-13 differentiate into the M2 phenotype during tumor progression and secrete IL-4, IL-5, and IL-6 to enhance angiogenesis, immunosuppression, and matrix remodeling (60). TAM cells secrete epidermal growth factor (EGF) and TNF-α, while tumor cells secrete colony-stimulating factor-1 (CSF-1). TAMs and tumor cells interact with each other to control the migration of cells in the microenvironment (61). TAMs can also promote cancer cell invasion by augmenting c-Jun and NF-κB activity as well as upregulating scavenger receptor A (SR-A) expression in ovarian cancer (62, 63). The stemness of ovarian cancer cells can be induced by coculturing these cells with macrophages (64). Conversely, cytokine and chemokine production derived from ovarian cancer cells can also affect macrophages by promoting their recruitment and changing their polarization. For example, LIF and IL-6 derived from ovarian cancer ascites can promote monocyte differentiation into M2 macrophages (65). EOC cells release CCL2, a chemokine, and CCL2/MCP-1 can recruit and polarize monocytes to TAMs in the TME (66). Ovarian cancer cell lines and tumor biopsy specimens express TNF, CCL22, and CXCL12, which induce macrophage polarization into the M2 phenotype in the TME (67). Periostin from ovarian cancer cells was shown to be a key factor in M2 macrophage recruitment (68). SEMA4D is a member of the transmembrane or secretory signaling protein IV subfamily. SEMA4D expression was higher in ovarian cancer cell lines and supernatant than in primary cultured human ovarian cells and supernatant. After stimulation with human recombinant soluble SEMA4D protein, peripheral blood monocytes tended to polarize into M2 macrophages (69). The polarization of M2 macrophages can also be regulated by COX-2 derived from ovarian cancer stem-like cells, which can activate the JAK and COX-2/PGE2 pathways (70).

Macrophages also play special roles in different histological subtypes of ovarian carcinoma. TAM infiltration was reported to be more frequent in ovarian serous and mucinous carcinoma than in other histological subtypes of ovarian cancer, and M2 macrophage infiltration in ovarian serous carcinoma indicates a poor prognosis (71, 72). Low- and high-grade serous ovarian cancers account for ~70% of all epithelial ovarian tumors and for a majority of deaths. Ciucci et al. (73) found that compared to high-grade serous ovarian cancer patients, low-grade patients had a lower density of tumor-infiltrating CD68+ macrophages and a subdued M2-skewed (CD163+) phenotype. This previous result may indicate that the differential activation of M2 macrophages may stimulate the development and spread of different subsets of ovarian cancer. In women, smoking increases the risk of only the mucinous subtype of ovarian cancer (74). Cigarette smoke can also activate nicotinic acetylcholine receptors, and this activation event has been demonstrated to polarize macrophages into the M2 phenotype (75). The relationship among smoking, macrophage polarization and ovarian mucinous cancer requires more research and discussion. Most ovarian endometrioid and clear cell carcinomas are caused by endometriosis (76). One study considered the possibility that CDC42-positive macrophages may inhibit the transformation of endometriosis into ovarian endometrioid and clear cell carcinomas (77). Glypican-3, which is specifically expressed in ovarian clear cell carcinoma, can increase the proportion of M1 macrophages and suppress the growth of mouse ovarian tumors (78). B7-H4 expressed on the surface of ovarian carcinoma cells is inversely associated with the infiltration of T cells and CD14+ macrophages in ovarian clear cell carcinoma but not serous or endometrioid carcinoma (79). Currently, studies on the role of macrophages in ovarian cancer subtypes are insufficient, and more studies are needed to explore more important functions of different types of macrophages in various ovarian cancer subtypes.

However, many studies have focused on the antitumor influences of M1 macrophages; interestingly, Untack Cho et al. showed that M1 macrophages promote metastasis in ovarian cancer by activating the NF-κB signaling pathway (80). These findings suggest that as macrophages are a member of the immune cell population in the cancer microenvironment, macrophage polarization plays a key role in the development, progression, and prognosis of ovarian cancer.

## Targeting Treatment to Macrophages in Ovarian Cancer

Cancer-related inflammation is one of the hallmarks of cancer, and some evidence shows that an inflammatory microenvironment promotes chemoresistance and genetic instability in tumor epithelial cells while also affecting resident or infiltrating immune cells, including macrophages (81, 82). These studies suggest that TAMs act as a “bridge” or mediator during the initiation and promotion of cancers by interacting with cancer cells. Four cancer therapy strategies involving tumoricidal effectors acting on TAMs exist: disturb TAM cell survival, inhibit the recruitment of macrophages, repolarize M2-like TAMs to M1-like macrophages, and deliver drugs with nanoparticle and liposome-based systems (83). It has been demonstrated that human recombinant antibodies (scFv) can be used to block mesothelin in combination with macrophages, which prevents the cancer-induced polarization of CD206^low^ macrophages toward the TAM phenotype. In addition, potential therapeutic agents for ovarian cancers that control the polarization of tumor-infiltrating innate immune cells have also been developed (84). Currently, some therapeutic drugs targeting TAMs are available for clinical use and experimental treatments. For example, the agent trabectedin interferes with the survival of TAMs (85), and alemtuzumab lowers the number of TAMs by targeting a surface protein on TAMs (86). Polymer nanoparticles loaded with cisplatin can be engulfed by TAMs and then act on cancer cells (87). By promoting antitumor immune responses and vessel normalization, host-produced histidine-rich glycoprotein (HRG) has been demonstrated to inhibit the growth and metastasis of tumors by controlling the polarization of TAMs from the M2 to the M1 phenotype (88). Drugs targeting macrophages could be useful in the treatment of ovarian cancer. Paclitaxel, an antitumor agent, is used to treat ovarian cancer and can reduce tumor growth by polarizing M2 into M1 macrophages in a TLR4-dependent manner (89). Some research has reported that the relationship between macrophage polarization and ovarian cancer is influenced by platinum. This study found that macrophages induced epithelial-to-mesenchymal transition (EMT) and the expression of some EMT genes in cisplatin-sensitive cells, while this response did not occur in cisplatin-resistant cancer cells (90).

TAMs also express immune checkpoint molecules, including B7-H4 and PD-L1, in ovarian cancer cells, which causes T cell exhaustion and an inactivated cytotoxic T cell response. High B7-H4 expression on the TAM surface correlates with high grades of ovarian cancer and poor survival rates for ovarian cancer patients (91). The use of some PDL-1 and PD-1 antibodies has been studied clinically in ovarian cancer patients. For example, in a clinical trial on the use of nivolumab as an anti-PD-1 antibody in platinum-resistant ovarian cancer patients, the disease control rate was 45%, and the median OS time was 20.0 months (UMIN Clinical Trials Registry UMIN000005714) (92). Authorization of pembrolizumab, another type of anti-PD-1 antibody, for the treatment of metastatic non-small-cell lung cancer has been accelerated by the Food and Drug Administration (FDA). A phase Ib study on pembrolizumab was performed using PDL-1-expressing advanced ovarian cancer patients, revealing that the safety and toxicity of its antitumor activity were manageable, and a phase II trial on pembrolizumab is ongoing (NCT02054806) (93). Furthermore, several clinical trials involving ovarian cancer patients are focused on the combined use of PDL-1/PD-1 antibodies and poly-ADP-ribose polymerase inhibitors (PARPi) or VEGF inhibitors; for example, the combinations of pembrolizumab and niraparib (NCT02657889), nivolumab and bevacizumab (NCT02873962), nivolumab and rucaparib (NCT03522246), and atezolizumab and bevacizumab (NCT038100) are being studied. CSF-1R is expressed in macrophages in ovarian cancer, and some clinical trials on antibodies and inhibitors of CSF-1R in addition to PDL-1 or PD-1 antibodies are also occurring (NCT02452424, NCT02526017, NCT02718911).

Results of a phase Ib clinical trial involving the combined use of a CCL2 antibody and four chemotherapy strategies for the treatment of solid tumor patients showed that carlumab, a CCL2 antibody, could be safely used (10 or 15 mg/kg) in combination with standard chemotherapy and had a good tolerating effect (NCT01204996). In addition, some natural plant products can inhibit tumor growth by changing the polarization of macrophages. For example, neferine from green seed lotus embryos exerts an antitumor effect on angiogenesis by regulating the polarization of TAMs in ovarian cancer (94). Deoxyschizandrin, a phytochemical, from berries can inhibit the activity of M2 macrophages, and onionin A not only increases cytotoxicity against ovarian cancer cells but also suppresses the activation of M2 macrophages (95). TAM repolarization can also be mediated by the natural compound baicalin (96).

## Outlook of Macrophage Polarization in Ovarian Cancer

### Noncoding RNAs

Noncoding RNAs are transcripts with no protein-coding capacity, and microRNAs (miRNAs) are small noncoding RNAs that are ~22–25 nucleotides long and bind to 3′-untranslated regions to inhibit gene expression at the posttranscriptional level by targeting mRNAs for cleavage or suppressing the expression of proteins (97). miRNAs have been implicated in many biological processes related to cell proliferation, differentiation, carcinogenesis, chemoresistance and metabolism (98, 99), and some miRNAs are also expressed in polarized macrophages. For example, miR-125a/b, miR-155, let-7e, and miRNA-378, induced by LPS and IFN-γ, are engaged in M1 phenotype polarization, while miR-223, induced by LPS, suppresses the activation of M1 macrophages. Some miRNAs, including miRNA-9, miRNA-21, miRNA-146, and miRNA-147, take part in M1 polarization via forming a negative feedback loop by interacting with NF-κB (30). miRNA-9, miRNA-21, miRNA-146, miRNA-147, miRNA-187, and miRNA-let-7c are involved in M2 polarization (100). M1 macrophages (classical activation) are characterized by microbicidal and tumoricidal activity, while M2 macrophages (alternative activation) are characterized by tumor progression and tissue remodeling (101). miRNAs regulate not only gene expression (via mRNA degradation) but also transcription factors in macrophage polarization (102). Long noncoding RNAs (lncRNAs), once regarded as “transcriptional noise,” range in length from 200 to 100,000 nucleotides (103) and can be located in the nucleus or cytoplasm. With the increasing knowledge regarding lncRNAs, they are now recognized as important factors in a variety of biological and pathological activities, including cancer processes, and have been demonstrated to impair the function and development of monocyte macrophages (104). Reduction in the expression of the lncRNA GAS5 has previously been linked to M2b macrophage polarization (105), and the lncRNA PCA3 promotes EOC tumorigenesis by disrupting gene expression by sponging miR-106b (106). Circular RNAs (circRNAs) form covalently closed loops by linking the 3′ and 5′ ends during RNA splicing (107, 108). Because of this loop structure, circRNAs are more stable than circulating tumor DNAs and linear RNAs in tissues, serum, saliva, and urine. As reported, circRNA molecules in eukaryotes derive from splicing, a cellular process mediated by the spliceosome machinery or by group I and group II ribozymes (108). CircRNAs are also widely associated with physiological and pathological processes, as they bind to RNA, RNA-binding proteins (RBPs), and translated peptides (109). A previous study showed that 189 circRNAs are differentially expressed between M1 and M2 macrophages (110). In cancers, circRNAs may regulate cell growth by sponging multiple miRNAs and changing the polarization of M1 and M2 macrophages (110, 111). Elucidating the roles of noncoding RNAs in macrophage polarization in ovarian cancers will provide promising information for the early diagnosis of tumors, evaluation of treatment, prediction of prognosis, and identification of potential targets for gene therapy in ovarian cancer.

### Exosomes

Exosomes, ~40–100 nm in size, are small membrane-bound vesicles that originate from multivesicular bodies (MVBs) and exist in extracellular fluids, such as the blood, cerebrospinal fluid, urine, amniotic fluid, seminal fluid, malignant ascites, breast milk, and saliva (112). As vehicles for intercellular communication, exosomes can transfer proteins, lipids, genomic materials and bioactive molecules, such as phosphatidyl-serine (PS), glycans, and glycoproteins (113, 114). Therefore, they have comprehensive biological functions. Compared with normal cells, tumor cells can more vigorously secrete exosomes and remodel immune cells to promote tumor initiation, invasion and metastasis by secreting exosomes into the TME (115). Exosomal noncoding RNAs, such as lncRNAs (116), microRNAs (117), and circRNAs (118), could be promising noninvasive biomarkers for ovarian cancers. EOC secretion of exosomal miR-222-3p can induce macrophages to polarize into a TAM-like phenotype (119). According to the functions of cargo molecules, exosomes can also promote the invasion of ovarian cancer (120). Exosome-mediated macrophage reprogramming to the M1 phenotype may be a promising therapy for cancer (121), and exosomes can also be predictors of treatment effectiveness and prognosis in ovarian cancer patients.

### Epigenetic Regulation

Epigenetic regulation does not change the genetic code but does control how information is encoded by DNA (122). The mechanisms of epigenetics are also mediated by posttranslational modifications, including methylation, acetylation, phosphorylation, β-N-glycosylation, and carbonylation of histones that bind DNA (123). The epigenetic markers histone modification and DNA methylation (DNAm) are reportedly more studied than the others. Histone modifications can function as epigenetic markers of the chromatin state and are related to multifarious macrophage survival, differentiation and activation processes (124, 125). The acetylation and deacetylation of histone proteins are achieved by histone acetyltransferases (HATs) and histone deacetylases (HDACs), and abnormal expression of HDACs has been found in many cancer types (126). HDAC3 can act as a brake for M2 polarization while promoting M1 responses (127). HDACs are a promising cancer treatment target, and more researchers are encouraged to study and develop HDAC inhibitors in clinical research. DNAm alters the expression of M1/M2 genes (128), and miRNAs can also be integrated by DNAm, contributing to macrophage heterogeneity and tumor processes. For instance, hypomethylated CpG sites with abnormal miRNA expression are associated with monocyte aging at CpG sites in the regions of the miR-29b-2, NRP1, and NRXN2 genes (129). The epigenetic silencing of miRNA expression by DNA hypermethylation promotes ovarian cancer aggressiveness (130). The functions of DNA methyltransferases (DNMTs) catalyze not only epigenetic silencing but also inappropriate activation of gene expression by DNAm, and DNMT1 and DNMT3b can mediate M1 macrophage polarization. The expression of Reprimo (RPRM) can be inhibited by DNMTs, and the RPRM tumor suppressor function can be restored by treatment with DNAm inhibitors (131). Sergey K et al. reported the development of a novel therapeutic strategy for methyltransferase by inhibiting the histone methyltransferase EZH2 in CARM1-expressing EOC (132). A DNAm inhibitor that resensitizes patients to traditional chemotherapy has been tested in a completed phase II clinical trial aimed at recurrent ovarian cancer patients (133). Changes in macrophage polarization and functional states need to accurately regulate the expression of target genes, which can be accomplished by epigenetic modifications (134).

## Conclusion

Because of the absence of curative treatment for advanced stages and relapsed disease, new therapeutic strategies for ovarian cancer are urgently needed. There are many classifications of ovarian cancer tissues. Currently, the suppression and eradication of cancer cells by activation of the innate immune system has shown inspiring results in some cancer treatments. As an important member of the TME infiltrating immune cell population, macrophages participate in the development and progression of ovarian cancers. In most cases, M1 macrophages play a role in antitumor immunity, while M2-like TAMs play a role in immunosuppression and tumor immune escape. The regulatory mechanisms of macrophage polarization may be contrary to one another. The work summarized in this review elaborates on the important roles of macrophage polarization in the development and progression of ovarian cancers. The polarization of macrophages is not only connected to the development, progression and prognosis of ovarian cancers but also provides some strategies for macrophage-centered ovarian cancer treatment to improve long-term survival.

## Author Contributions

HC drafted the manuscript. ZW and LF revised the manuscript. TX designed the topic and revised the manuscript. All authors read and approved the final manuscript.

### Conflict of Interest Statement

The authors declare that the research was conducted in the absence of any commercial or financial relationships that could be construed as a potential conflict of interest.

## Abbreviations

OvCa: Ovarian cancer
TAM: Tumor-associated macrophage
PFS: progression-free survival
OS: overall survival
ECM: Extracellularmatrixc
TME: Tumor microenvironment
HRG: Host-produced histidin-rich glycoprotein
FcR: Fc receptor
IC: Immune complex
IFN-g: Interferon-g
iNOS: Inducible nitric oxide synthase
LPS: Lipopolysaccharide
MR: Mannose receptor
RNI: Reactive nitrogen intermediates
ROI: Reactive oxygen intermediates
SLAM: Signaling lymphocytic activation molecule
SRs: S cavenger receptors
TLR: Toll-like receptor
MHC II: Major histocompatibility complex class II
TGF-β: Tumor growth factor-β
Arg-1: Arginase-1
FIZZ1: Resistin-like molecule-alpha (Relm-alpha)
GCs: Glucocorticoids
IL1-ra: IL-1 receptor antagonist
LIF: Leukocyte inhibitory factor
TGM2: Transglutaminase 2
TNF-α: tumor necrosis factor α
TLR: Toll-like receptor
MMR: (CD206) macrophage mannose receptor
SOCS3: Suppressor of cytokine signaling 3
VEGF: Vascular endothelial growth factor
Ym1: (Chi3l3)chitinase-3-like protein-3
JAK: Janus Kinase
STAT: Signal transducers and activators of transcription
JNK: c-Jun N-terminal kinase
AMPK: Adenosine 5'-monophosphate (AMP)-activated protein kinase
IL: interleukin
PPARγ: Peroxisome proliferator-activated receptor γ
ACE: Angiotensin-converting enzyme
PIP3, phosphatidylinositol 3, 4: 5-tris phosphate
NF-κB: Nuclear factor-κB
C/EBP: CCAAT-enhancer-binding protein
LIF: Leukocyte inhibitory factor
HATs: Histone acetyltransferases
HDACs: Histone deacetylases
EGF: Epidermal growth Factors
CSF-1: Colony-stimulating factor-1
LIF: Leukemia inhibitory factor
EMT: Epithelial-mesenchymal transition
EOC: Epithelial ovarian cancer
PARPi: Poly-ADP-ribose polymerase inhibitors
VEGF: Vascular endothelial growth factor receptor
MiRNA: MicroRNAs
lncRNAs: Long non-coding RNAs
CircRNAs: Circular RNAs
MVBs: Multivesicular bodies
scFv: Human recombinant antibodies
PD-1: Programmed cell death protein-1
PD-L1: Programmed cell death-Ligand 1
SR-A: Scavenger receptor A.
